# Predicting the Thermodynamic Ideal Glass Transition Temperature in Glass-Forming Liquids

**DOI:** 10.3390/ma13092151

**Published:** 2020-05-06

**Authors:** Qian Gao, Zengyun Jian

**Affiliations:** School of Materials and Chemical Engineering, Xi’an Technological University, Xi’an 710021, China; xian1504@126.com

**Keywords:** glass transition temperature, fragility parameter, Kauzmann temperature, thermodynamic ideal glass transition temperature, glass-forming liquid

## Abstract

The Kauzmann temperature *T*_K_ is a lower limit of glass transition temperature, and is known as the ideal thermodynamic glass transition temperature. A supercooled liquid will condense into glass before *T*_K_. Studying the ideal glass transition temperature is beneficial to understanding the essence of glass transition in glass-forming liquids. The Kauzmann temperature *T*_K_ values are predicted in 38 kinds of glass-forming liquids. In order to acquire the accurate predicted *T*_K_ by using a new deduced equation, we obtained the best fitting parameters of the deduced equation with the high coefficient of determination (*R*^2^ = 0.966). In addition, the coefficients of two reported relations are replaced by the best fitting parameters to obtain the accurate predicted *T*_K_, which makes the *R*^2^ values increase from 0.685 and 0.861 to 0.970 and 0.969, respectively. Three relations with the best fitting parameters are applied to obtain the accurate predicted *T*_K_ values.

## 1. Introduction

**Figure 1:**
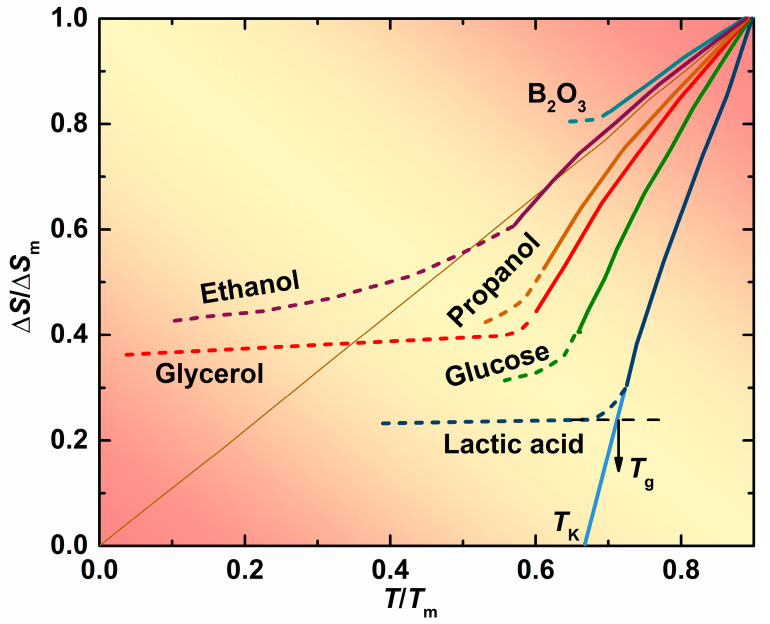
Temperature dependence of the difference in entropy between various supercooled liquids and their crystalline phases. Δ*S*_m_ and *T*_m_ are the melt entropy and the melting temperature, respectively. (Adapted from Ref. [5,7]).

If crystallization can be avoided by sufficiently rapid cooling, a supercooled liquid will become a glassy state at glass transition temperature *T*_g_, at which the viscosity of the supercooled liquid is typically 10^12^ Pa s (10 poise = 1 Pa s) [1,2,3,4,5]. Liquid–glass transitions are generally observed in various supercooled liquids, including molecular liquids, ionic liquids, metallic liquids, oxides, and chalcogenides [5,6]. Figure 1 shows the temperature dependence of the entropy difference between various supercooled liquids and their crystalline phases [5,7]. With temperature decreases, their entropic surplus is consumed, and the glass transition sets in when the slope of the curve changes. For lactic acid, its glass transition temperature *T*_g_ has been marked in Figure 1. Its curve can then be extrapolated to the Kauzmann temperature *T*_K_, at which point Δ*S* will vanish. In other words, the entropy of the supercooled liquid equals the entropy of its crystalline counterpart at *T*_K_. Below *T*_K_, the entropy of the supercooled liquid will become less than that of its crystalline phase. However, it is difficult to see how a disordered and nonperiodic liquid has a lower entropy than a periodic crystal of the same density [8]. As a consequence, Kauzmann temperature *T*_K_ is a lower limit of glass transition temperature (i.e., the thermodynamic ideal glass transition temperature) and the supercooled liquid will condense into glass, having the same entropy as the perfect crystal at *T*_K_ [8].

The glass transition temperature *T*_g_ plays an important role in liquid–glass transition. *T*_g_ has significant thermophysical properties for predicting glass-forming ability (GFA) and the stability of glass formers. Thermodynamically, the lowest value of *T*_g_ is the Kauzmann temperature *T*_K_ for a certain glass-forming liquid. In other words, the Kauzmann temperature *T*_K_ is the lowest temperature at which a supercooled liquid can exist. The Kauzmann temperature *T*_K_ is studied, which is beneficial to understand the nature of glass transition, and to find a correlation between the measured glass transition temperature *T*_g_ and the thermodynamically ideal glass transition temperature *T*_K_. In addition, it can be seen that fragility parameter *m* is related to *T*_g_ and *T*_K_ (see below). The fragility parameter *m* is applied to describe the degree of departure from an Arrhenius relation of the temperature dependence of viscosity. That is, *T*_g_ and *T*_K_ can also be applied to describe the temperature dependence of viscosity in glass-forming liquids. Therefore, studying the temperature *T*_K_ is a classic problem in amorphous materials. *T*_K_ temperatures in various glass-forming liquids have been calculated, such as in metallic liquids [4,9,10,11,12,13,14,15,16], molecular liquids [6,17], ionic liquids [6,17], and oxides [6,12]. The Kauzmann temperature *T*_K_ will be predicted by *T*_g_ and *m* in glass-forming liquids.

## 2. Expressions of Predicting *T*_K_

Universally, *T*_K_ can be acquired by [5,9,11,14,18]:

(1) $$\Delta S_{m}=\int_{T_{K}}^{T_{m}}\frac{\Delta c_{p}^{l-c}(T)}{T}dT$$

where Δ*S*_m_ is the entropy of fusion at the melting point *T*_m_, and Δ*c*_p_^l-c^(*T*) is the specific heat capacity difference between the supercooled liquid and its crystalline counterpart. If Δ*S*_m_ and Δ*c*_p_^l-c^(*T*) are acquired, *T*_K_ will be calculated. The entropy of fusion Δ*S*_m_, can be obtained by Δ*S*_m_=Δ*H*_m_/*T*_m_, where Δ*H*_m_ is the heat of fusion, which can be obtained by the integration of the melting peak [19]. The so-called “step method”, which consists of heating the sample to a certain temperature with a constant rate, and then annealing isothermally during each step, can be applied to determine the specific heat capacity of the sample on heating, in reference to the specific heat capacity of a standard sapphire [4,15]. The data of *c*_p_(*T*)_sample_ can be calculated by the following equations [4,15]:

(2) $$c_{p}{\left(T\right)}_{\mathrm{sample}}=\frac{Q_{sample}^{*}-Q_{pan}^{*}}{Q_{sapphire}^{*}-Q_{pan}^{*}}\times \frac{m_{\mathrm{sapphire}}\times \mu_{\mathrm{sample}}}{m_{\mathrm{sample}}\times \mu_{\mathrm{sapphire}}}\times c_{p}{\left(T\right)}_{\mathrm{sapphire}}$$

where *c*_p_(*T*)_sample_ and *c*_p_(*T*)_sapphire_ are the specific heat capacity of sample and sapphire, respectively, *m_i_* the mass, *μ_i_* the mole mass, and $Q_{i}^{*}$ the heat flux. Meanwhile, the temperature dependence of the specific heat capacity *c*_p_^liquid^(*T*) of the supercooled liquid can be expressed as [4,11,15]:

(3) $$c_{p}(T)=3R+a\cdot T+b\cdot T^{-2}$$

where *R* is gas constant. The specific heat capacity *c*_p_^crystal^(*T*) of the crystal can be expressed as [4,11,15]:

(4) $$c_{p}(T)=3R+c\cdot T+d\cdot T^{2}$$

The parameters of expressions for *c*_p_^liquid^(*T*) and *c*_p_^crystal^(*T*) can be determined by fitting the data measured in steps in reference to sapphire. Therefore, the specific heat capacity difference between the supercooled liquid and its crystalline counterpart can be calculated by Equations (3) and (4), with the known parameters. *T*_K_ can be calculated by the above formulas so far. From the above analysis, acquiring the Kauzmann temperature *T*_K_ is cumbersome and time-consuming. Therefore, the easily obtained parameters are applied to predict *T*_K_.

The Angell’s fragility parameter *m*, based on viscosity or relaxation time, is defined as [1,20,21,22,23]:

(5) $$m=\frac{d\mathrm{lg}(\eta )}{d(T_{g}/T)}|{}_{T=T_{g}}=\frac{d\mathrm{lg}(\tau )}{d(T_{g}/T)}|{}_{T=T_{g}}=\frac{DT_{0}T_{g}}{{\left(T_{g}-T_{0}\right)}^{2}\ln(10)}$$

A similar fragility has been defined as [17]:

(6) $$m_{S}=\frac{d\left[\frac{\mathrm{lg}(\eta (T))/\eta_{0}^{}}{\mathrm{lg}(\eta (T_{g})/\eta_{0})}\right]}{d(T_{g}/T)}|{}_{T=T_{g}}=\frac{m}{m_{\min}}$$

where *m*_min_ = log_10_(*η*_g_/*η*_0_). *η*_g_ denotes viscosity (typically 10^12^ Pa s) at glass transition temperature *T*_g_. *η*_0_ is the high temperature limit of viscosity, which can be determined by the following equation [2,24]:

(7) $$\eta_{0}=hN_{A}\rho /M$$

where *h* is Planck’s constant, *N*_A_ is Avogadro’s number, *ρ* is the density of the liquid and *M* is the molar mass. The *η*_0_ value is about set as 10^−5^ Pa s [2,17,24,25,26]. Thus, generally, the log_10_(*η*_g_/*η*_0_) value is equal to 17. The expressions related to *T*_K_ have been studied, and they can be utilized to calculate *T*_K_, which make calculation simpler. *T*_K_ as a function of *T*_g_ and Angell’s fragility parameter *m* has been reported, and the expression can be described by [1,16,17,25]:

(8) $$m_{s}=\frac{m}{m_{\min}}=\frac{T_{g}^{}}{T_{g}^{}-T_{K}^{}}$$

From Equation (8), *T*_K_ can be expressed as:

(9) $$T_{K}=T_{g}-m_{\min}T_{g}/m$$

The other expression of *T*_K_ as a function of *T*_g_ and *m* can be expressed as [1,16,17,25]:

(10) $$m_{s}=\frac{m}{m_{\min}}=\frac{T_{g}^{2}+T_{K}^{2}}{T_{g}^{2}-T_{K}^{2}}$$

Equation (10) is transformed into:

(11) $$T_{K}=T_{g}\times {\left[(m-m_{\min})/(m+m_{\min})\right]}^{1/2}$$

Furthermore, a new expression of predicting *T*_K_ as a function of *T*_g_ and *m* is also deduced by us, and this expression is derived as follows. Another expression of the Kauzmann temperature is presented by [27]:

(12) $$T_{K}=T_{m}{\left(1+\frac{\Delta H_{m}}{T_{g}\Delta c_{p}^{l-c}(T_{g})}\right)}^{-1}$$

Additionally, *m* can be calculated by the following Equation [27]:

(13) $$m_{}=\Lambda_{a}\frac{\Delta c_{p}^{l-g}(T_{g})}{\Delta S_{m}}$$

where *Λ*_a_ is the constant and equals 40. Δ*c*_p_^l-g^(*T*_g_)=*c*_p_^liquid^(*T*_g_)-*c*_p_^glass^(*T*_g_) is the specific heat capacity difference between the supercooled liquid and its glass state at *T*_g_. When Δ*c*_p_^l-g^(*T*_g_) is replaced by Δ*c*_p_^l-c^(*T*_g_), the numerical factor would increase from 40 to 43, but the quality of the correlation remains unchanged, where Δ*c*_p_^l-c^(*T*_g_)=*c*_p_^liquid^(*T*_g_)-*c*_p_^crystal^(*T*_g_) is the specific heat capacity difference between the supercooled liquid and its crystalline counterpart at *T*_g_ [27]. Hence, Δ*c*_p_^l-g^(*T*_g_) is replaced by Δ*c*_p_^l-c^(*T*_g_), and *m* can be expressed as:

(14) $$m=\Lambda_{b}\frac{\Delta c_{p}^{l-c}(T_{g})}{\Delta S_{m}}$$

where *Λ*_b_ is the constant and equals 43. The ratio *T*_m_/*T*_g_ is about constant *Λ*_c_, which equals 3/2 [27,28,29,30]. Plugging this *T*_m_/*T*_g_ relation into Equation (14):

(15) $$m=\Lambda_{b}\Lambda_{c}T_{g}\frac{\Delta c_{p}^{l-c}(T_{g})}{\Delta H_{m}}$$

From Equation (12) and Equation (15), we obtain:

(16) $$T_{K}=\Lambda_{c}T_{g}{\left(1+\frac{\Lambda_{b}\Lambda_{c}}{m_{}}\right)}^{-1}=\Lambda_{c}T_{g}(\frac{m}{m_{}+\Lambda_{b}\Lambda_{c}})$$

The expanded Equation (16) can be expressed by:

(17) $$T_{K}=\Lambda_{c}T_{g}-\Lambda_{b}\Lambda_{c}^{2}\frac{T_{g}}{m_{}+\Lambda_{b}\Lambda_{c}}$$

It can be seen that these expressions of predicting *T*_K_ are expressed as the function of *T*_g_ and *m* from Equations (9), (11), and (17). Because *T*_g_ and *m* have been reported for a lot of glass-forming liquids, predicting *T*_K_ will be made simpler and more convenient by the above formulae.

## 3. Methods

As can be seen from the above, Equations (9), (11), and (17) can be applied to predict *T*_K_. In order to obtain accurate *T*_K_ values, the coefficient of determination, *R*^2^ is applied to evaluate the accuracy of the predicted *T*_K_. In statistics, generally, *R*^2^ is defined as: *R*^2^ = 1−SS_res_/SS_tot_, where SS_res_ is the sum of squares of residuals and SS_tot_ is the total sum of squares. *R*^2^ is a statistical measure of how well the predicted *T*_K_ values approximate the reported *T*_K_ values. The higher is the *R*^2^ value (0 ≤ *R*^2^ ≤ 1), the more accurate is the predicted *T*_K_. The predicted *T*_K_ values perfectly fit the reported *T*_K_ when *R*^2^ equals 1.

## 4. Results and Discussion

**Figure 2:**
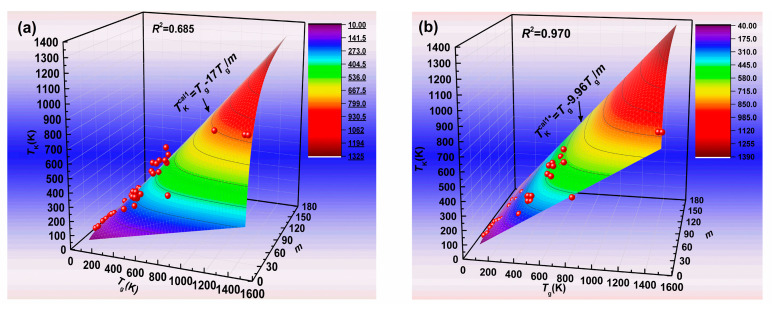
The predicted Kauzmann temperature obtained by Equation (9). (**a**) *m*_min_ = 17; (**b**) *m*_min_ = 9.96.

The values of the glass transition temperature *T*_g_, Angell’s fragility parameter *m*, and the Kauzmann temperature *T*_K_ for various glass formers are listed in Table 1. Figure 2a shows the predicted Kauzmann temperature *T*_K_^cal1^, according to Equation (9) at *m*_min_ = 17. Many reported *T*_K_ values for various glass formers do not fall on the curve of the predicted *T*_K_^cal1^. Meanwhile, the *R*^2^ value of this correlation equals 0.685, which is relatively low. It indicates that the predicted *T*_K_^cal1^ values by using Equation (9) at *m*_min_ = 17 are inaccurate. Although the log_10_(*η*_g_/*η*_0_) (i.e., *m*_min_) value is generally equal to 17, the viscosity change in the glass transition is approximately two orders of magnitude [16,18]. Therefore, the log_10_(*η*_g_/*η*_0_) value is considered to have a range from 15 to 17 [16]. In fact, generally, the *η*_0_ value is set as about 10^−5^ Pa s, but *η*_0_ values have differences in some amorphous materials [31]. This will cause a change of the log_10_(*η*_g_/*η*_0_) value as well. In our previous study, the log_10_(*η*_g_/*η*_0_) value is considered to have a range from 14 to 18 [32]. As a result, the *m*_min_ value slightly fluctuates. In order to obtain the most accurate predicted Kauzmann temperature by using Equation (9), we regard the *m*_min_ value as a fitting parameter, which has no restrictions, and can be an arbitrary value. Therefore, we obtain the best fit and the most accurate predicted Kauzmann temperature *T*_K_^cal1*^ by using Equation (9), when *m*_min_ equals 9.96. Although there is a difference between this value (*m*_min_ = 9.96) and the *m*_min_ value obtained by *η*_g_ and *η*_0_ of the amorphous materials, and this value may not have a physical meaning, the most accurate *T*_K_^cal1*^ by using Equation (9) at *m*_min_ = 9.96 can be obtained. Our purpose is to make the Kauzmann temperature accurately predictable, so it is feasible that the most accurate predicted Kauzmann temperature *T*_K_^cal1*^ values are obtained by using Equation (9) at *m*_min_ = 9.96. Figure 2b shows the predicted Kauzmann temperature *T*_K_^cal1*^, according to Equation (9) at *m*_min_ = 9.96. From Figure 2, it can be seen that the *R*^2^ value greatly increases from 0.685 to 0.970 when the predicted values obtained by using Equation (9) at *m*_min_ = 17 are replaced by those obtained by using Equation (9) at *m*_min_ = 9.96. It indicates that the accuracy of the predicted values obtained by using Equation (9) at *m*_min_ = 9.96 are greatly improved. The predicted values obtained by using Equation (9) at *m*_min_ = 17 and 9.96 have also been listed in Table 1 for convenience in comparing the predicted (*T*_K_^cal1^ and *T*_K_^cal1*^) values with the reported *T*_K_ values.

**Figure 3:**
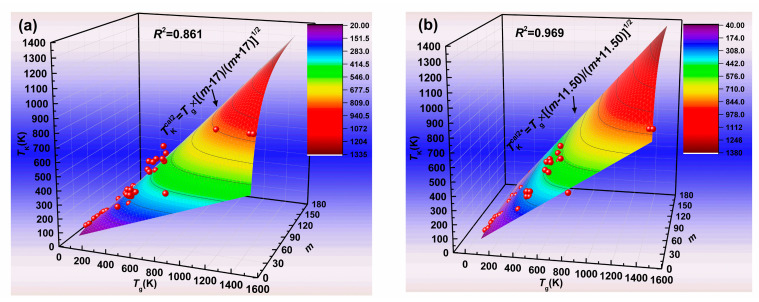
The predicted Kauzmann temperature obtained by Equation (11). (**a**) *m*_min_ = 17; (**b**) *m*_min_ = 11.50.

Figure 3a illustrates the predicted Kauzmann temperature *T*_K_^cal2^ by Equation (11) at *m*_min_ = 17. Compared to Figure 2a, the predicted Kauzmann temperature *T*_K_^cal2^ values (*R*^2^ = 0.861) obtained by Equation (11) at *m*_min_ = 17 are more accurate than those obtained by Equation (9) at *m*_min_ = 17. In order to obtain the most accurate predicted Kauzmann temperature by using Equation (11), we also regard the *m*_min_ value as the fitting parameter. Therefore, we obtain the best fit and the most accurate predicted Kauzmann temperature *T*_K_^cal2*^ by using Equation (11), when *m*_min_ equals 11.50. Figure 3b shows the predicted Kauzmann temperature *T*_K_^cal2*^, according to Equation (11) at *m*_min_ = 11.50. From Figure 3, it can be seen that the *R*^2^ value increases from 0.861 to 0.969 when the predicted values obtained by using Equation (11) at *m*_min_ = 17 are replaced by those obtained by using Equation (11) at *m*_min_ = 11.50. The predicted values obtained by using Equation (11) at *m*_min_ = 17, and 11.50 are also listed in Table 1.

**Figure 4:**
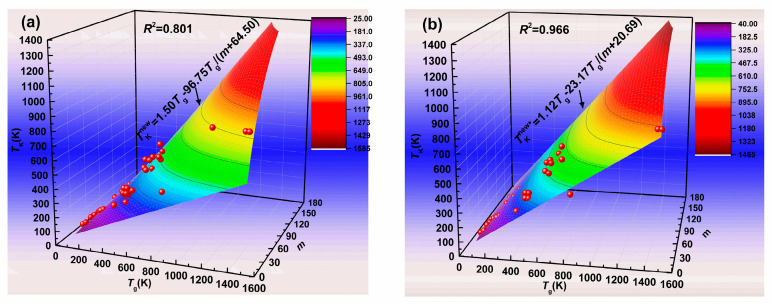
The predicted Kauzmann temperature obtained by Equation (17). (**a**) *Λ*_b_ = 43 and *Λ*_c_ = 1.5; (**b**) *Λ*_b_ = 18.47 and *Λ*_c_ = 1.12.

A new formula (Equation (17)) has been deduced in the above introduction, whose expression is also a function of *T*_g_ and *m*. In the literature, *Λ*_b_ equals 43 [27] and *Λ*_c_ (the ratio *T*_m_/*T*_g_) is equal to about 3/2 [27,28,29,30]. We plug these values into Equation (17), and the curve of the predicted *T*_K_^new^ is shown in Figure 4a. The *R*^2^ value of this correlation equals 0.801. In order to obtain the most accurate predicted Kauzmann temperature by using Equation (17), we also regard *Λ*_b_ and *Λ*_c_ values as the fitting parameters. The best fit of the experimental data yields *Λ*_b_ = 18.47 and *Λ*_c_ = 1.12. Plugging the fitted values into Equation (17):

(18) $$T_{K}^{\mathrm{new}*}=1.12T_{g}-23.17\frac{T_{g}}{m+20.69}$$

Figure 4b shows the predicted Kauzmann temperature *T*_K_^new*^, according to Equation (18). From Figure 4, it can be seen that the *R*^2^ value increases from 0.801 to 0.966 when the predicted values obtained by using Equation (17) at *Λ*_b_ = 43 and *Λ*_c_ = 3/2 are replaced by those obtained by using Equation (17) with the best fitting parameters (i.e., Equation (18)). The predicted values obtained by using Equation (17) at *Λ*_b_ = 43 and *Λ*_c_ = 3/2 and using Equation (18) are also listed in Table 1.

## 5. Conclusions

The Kauzmann temperature *T*_K_ in 38 kinds of amorphous materials have been predicted. Meanwhile, we regard the *m*_min_ value as the fitting parameter to improve the accuracy of predicting *T*_K_ values. The coefficient of determination *R*^2^ values increase from 0.685 and 0.861 to 0.970 and 0.969, respectively, when the coefficients of two reported relations are replaced by the best fitting parameters. In addition, a new formula of predicting *T*_K_ values with *R*^2^ = 0.966 is deduced. Therefore, three equations with the best fitting parameters have relatively high *R*^2^ values, which indicates that they can be applied to obtain the accurate predicted *T*_K_ values.

## Figures and Tables

**Table 1:** The values of *T*_g_, *T*_K_, and *m* for various glass-forming liquids. The data (numbers 19–38) were taken from Ref. [17,33].

	Glass Formers	*T*g (K)	*m*	*T*_K_ (K)	*T*_K_^cal1^ (K)	*T*_K_^cal1*^ (K)	*T*_K_^cal2^ (K)	*T*_K_^cal2*^ (K)	*T*_K_^new^ (K)	*T*_K_^new*^ (K)
1	Mg_65_Cu_25_Y_10_	404 [4]	50 [34]	320 [4]	266.64	323.52	283.53	319.65	264.63	320.06
2	Pd_77.5_Cu_6_Si_16.5_	637 [34]	73 [34]	560 [16,35]	488.66	550.09	502.47	543.44	507.28	555.91
3	Cu_47_Ti_34_Zr_11_Ni_8_	673 [34,36]	59 [34]	537 [36]	479.08	559.39	500.30	552.42	482.27	558.08
4	Zr_41.2_Ti_13.8_Cu_12.5_Ni_10_Be_22.5_	625 [34,37]	46 [34]	558 [6,13]	394.02	489.67	424.04	484.12	390.27	482.86
5	Zr_46.75_Ti_8.25_Cu_7.5_Ni_10_Be_27.5_	590 [13,34]	46 [34]	560 [13]	371.96	462.25	400.30	457.01	368.42	455.82
6	SiO_2_	1480 [38]	25 [34]	876 [38]	473.60	890.37	645.92	900.08	620.11	907.07
6	SiO_2_	1452 [34,38]	25 [34]	876 [38]	464.64	873.52	633.70	883.05	608.38	889.91
7	GeO_2_	816 [34,38]	21 [34]	418 [38]	155.43	428.98	264.75	441.17	300.63	460.41
8	Pd_40_Ni_40_P_20_	578 [3,13]	46 [3,13]	500 [9,13]	364.39	452.85	392.15	447.72	360.92	446.55
9	La55Al25Ni20	491 [13,34,39]	42 [34]	337 [10,13]	292.26	374.56	319.61	370.73	290.45	368.45
9	La55Al25Ni20	470.3 [10]	42 [34]	337 [10,13]	279.94	358.77	306.14	355.10	278.21	352.91
10	La55Al25Ni15Cu5	472 [13,34,39]	37 [34]	318 [10,13]	255.14	344.94	287.25	342.25	258.09	339.07
10	La55Al25Ni15Cu5	449.3 [10]	37 [34]	318 [10,13]	242.86	328.35	273.44	325.79	245.68	322.76
11	La55Al25Ni10Cu10	467 [13,34,39]	35 [34]	332 [10,13]	240.17	334.11	274.76	331.99	246.41	328.74
11	La55Al25Ni10Cu10	440.6 [10]	35 [34]	332 [10,13]	226.59	315.22	259.23	313.22	232.48	310.16
12	La_55_Al_25_Ni_5_Cu_15_	459 [13,34,39]	42 [34]	304 [10,13]	273.21	350.15	298.78	346.57	271.52	344.44
12	La_55_Al_25_Ni_5_Cu_15_	435 [10]	42 [34]	304 [10,13]	258.93	331.84	283.16	328.44	257.32	326.43
13	La_55_Al_25_Ni_5_Cu_10_Co_5_	466 [13,16,34,39]	37 [16,34]	363 [13,16]	251.89	340.56	283.60	337.90	254.81	334.76
13	La_55_Al_25_Ni_5_Cu_10_Co_5_	439.1 [10]	37 [16,34]	363 [13,16]	237.35	320.90	267.23	318.39	240.10	315.44
14	Zr_46_(Cu_4.5_/_5.5_Ag_1_/_5.5_)_46_Al_8_	703 [14]	49 [14,16]	671 [14,16]	459.10	560.10	489.51	553.47	455.25	553.63
15	Zr_46_Cu_46_Al_8_	715 [14,16]	43 [14,16]	596 [14,16]	432.33	549.39	470.67	543.58	429.00	540.69
16	Zr_44_Ti_11_Ni_10_Cu_10_Be_25_	620 [16,40]	39 [16]	504.5 [16]	349.74	461.66	388.61	457.52	350.43	453.73
17	Pd_43_Ni_10_Cu_27_P_20_	582 [15,16]	65 [12,16]	532 [15,16,41]	429.78	492.82	445.28	486.71	438.19	494.47
17	Pd_43_Ni_10_Cu_27_P_20_	576 [11]	65 [12,16]	447 [11,12]	425.35	487.74	440.69	481.69	433.67	489.37
18	Au_77_Ge_13.6_Si_9.4_	294 [16]	85 [12,16]	199 [16]	235.20	259.55	240.05	256.58	250.74	264.83
19	2-metylpentane	80.5	58	58	56.91	66.68	59.52	65.85	57.17	66.46
20	Butyronitrile	100	47	81.2	63.83	78.81	68.47	77.90	63.23	77.77
21	Ethanol	92.5	55	71	63.91	75.75	67.20	74.81	63.86	75.28
22	n-propanol	102.5	36.5	73	54.76	74.53	61.88	73.97	55.56	73.27
23	Toluene	126	59	96	89.69	104.73	93.67	103.42	90.29	104.49
24	1-2 propan diol	172	52	127	115.77	139.06	122.50	137.36	115.16	137.81
25	Glycerol	190	53	135	129.06	154.29	136.26	152.40	128.55	153.06
26	Triphenil phospate	205	160	166	183.22	192.24	184.26	190.76	219.15	203.31
27	Orthoterphenyl	244	81	200	192.79	214.00	197.18	211.50	203.75	217.68
28	m-toluidine	187	79	154	146.76	163.42	150.28	161.50	154.42	165.98
29	Propylene carbonate	156	104	127	130.50	141.06	132.28	139.61	144.43	145.73
30	Sorbitol	266	93	226	217.38	237.51	221.10	234.91	235.60	243.71
31	Selenium	307	87	240	247.01	271.85	251.87	268.78	264.45	277.79
32	ZnCl_2_	380	30	250	164.67	253.84	199.85	253.71	180.95	251.90
33	As_2_S_3_	455	36	265	240.14	329.12	272.43	326.77	244.48	323.64
34	CaAl_2_Si_2_O_8_	1118	53	815	759.40	907.90	801.76	896.78	756.43	900.63
35	Propilen glycol	167	52	127	112.40	135.01	118.94	133.37	111.81	133.81
36	3-Methyl pentane	77	36	58.4	40.64	55.70	46.10	55.30	41.37	54.77
37	3-Bromopentane	108	53	82.5	73.36	87.70	77.45	86.63	73.07	87.00
38	2-methyltetrahydrofuran	91	65	69.3	67.20	77.06	69.62	76.10	68.51	77.31
